# Acid sphingomyelinase inhibition alleviates muscle damage in gastrocnemius after acute strenuous exercise

**DOI:** 10.20463/jenb.2019.0009

**Published:** 2019-06-30

**Authors:** Young-Ik Lee, Yea-Hyun Leem

**Affiliations:** 1 Department of Oriental Sports Medicine, Daegu Haany University, Gyeongsan-si Republic of Korea; 2 Department of Molecular Medicine and Tissue Injury Defense Research Center, Ewha Womans University, Seoul Republic of Korea

**Keywords:** Strenuous exercise, muscle damage, creatine kinase, interleukin-6, sphingomyelinase, imipramine

## Abstract

**[Purpose]:**

Strenuous exercise often induces skeletal muscle damage, which results in impaired performance. Sphingolipid metabolism contributes to various cellular processes, including apoptosis, stress response, and inflammation. However, the relationship between exercise-induced muscle damage and ceramide (a key component of sphingolipid metabolism), is rarely studied. The present study aimed to explore the regulatory role of sphingolipid metabolism in exercise-induced muscle damage.

**[Methods]:**

Mice were subjected to strenuous exercise by treadmill running with gradual increase in intensity. The blood and gastrocnemius muscles (white and red portion) were collected immediately after and 24 h post exercise. For 3 days, imipramine was intraperitoneally injected 1 h prior to treadmill running.

**[Results]:**

Interleukin 6 (IL-6) and serum creatine kinase (CK) levels were enhanced immediately after and 24 h post exercise (relative to those of resting), respectively. Acidic sphingomyelinase (A-SMase) protein expression in gastrocnemius muscles was significantly augmented by exercise, unlike, serine palmitoyltransferase-1 (SPT-1) and neutral sphingomyelinase (N-SMase) expressions. Furthermore, imipramine (a selective A-SMase inhibitor) treatment reduced the exercise-induced CK and IL-6 elevations, along with a decrease in cleaved caspase-3 (Cas-3) of gastrocnemius muscles.

**[Conclusion]:**

We found the crucial role of A-SMase in exercise-induced muscle damage.

## INTRODUCTION

Sphingolipids, including ceramide (Cer), are important bioactive molecules that are involved in diverse biological processes, including apoptosis, proliferation, differentiation, growth arrest, and inflammation^1-5^. Cer, a central lipid moiety and a key secondary messenger of sphingolipid pathway, activates several kinases, phosphatases, and transcription factors^6-8^. Cellular Cer is produced by two major pathways, which includes de novo synthesis and sphingomyelin hydrolysis. In particular, Cer located in the plasma membrane primarily exerts a structural role under normal state, whereas, it possesses a pro-apoptotic property and increases cellular contents in response to stresses such as cytokines^9-12^. In vitro and animal studies have demonstrated Cer-induced lipotoxicity in cardiomyocytes. This is evidenced by the oxidative stress and cell death caused by the enhanced activity of ceramide synthase (-2 or -5)-provoked myocytic Cer accumulation^13-14^. Enhanced SPT-1 and sphingomyelinase (SMase) expressions leading to intracellular Cer accumulation along with age-related muscle weakness in gastrocnemius muscle of aged rats were observed^15^. Furthermore, the gastrocnemius muscle of aerobic exercised (5-week) rats exhibited enhanced activities of SPT and SMases, whereas that of ceramidase was reduced without alterations in Cer levels^16^. Based on this information, it is speculated that sphingolipid metabolism may contribute to exercise-induced skeletal muscle damage similar to strenuous exercise-produced cell death caused by sphingolipid metabolism and myocyte inflammation.

Exercise-induced muscle damage (EIMD) holds importance in the field of exercise and sports and is caused by unaccustomed or strenuous exercise involving a large amount of eccentric muscle contractions^17-19^. EIMD is characterized by ultra-structural myofibrillar disruption such as Z-line streaming, delayed onset muscle soreness (DOMS), secondary inflammation resulting from leukocyte infiltration, and systemic release of cellular enzymes and proteins such as creatine kinase (CK)^20-23^. As mentioned above, non-homeostatic skeletal muscle shift triggered by strenuous exercise, leads to impaired performance including decreased maximal force-generating capacity, elevated local muscle pain, and reduced training quality. Therefore, it is necessary to explore a cellular mechanism that regulates EIMD and to develop an efficient strategy for reducing EIMD for a rapid and successful repair and recovery process of the damaged muscle.

However, studies focusing on the direct relationship between exercise-induced muscle damage and sphingolipid metabolism remain limited. In the present study, we explored whether the rate-limiting enzymes of sphingolipid metabolism, such as SPT-1 and SMases contribute to EIMD in gastrocnemius muscle after acute strenuous exercise.

## METHODS

### Experimental mice and procedures

Seven week-old C57BL/6 male mice were acquired from Daehan Biolink, Co., Ltd. (Eumsung, Chungbuk, Korea) and housed in clear plastic cages under specific pathogen-free conditions and a 12:12 h light-dark cycle (light period: 08:00-20:00). The mice had free access to standard irradiated chow diet (Purina Mills, Seoul, Korea). All of the experimental procedures involving animals were approved by the Animal Care and Use Committee of Ewha Women’s University.

The mice were acclimatized to treadmill-running (Myung Jin Instruments Co., Seoul, Korea) by pre-exercising (Pre-Ex) at 10 m/min for 20 min/day (5% grade) for 3 days (each group, N = 9). The animals were placed on a restricted daily diet, 4 h prior to exercise. The protocol of exercise-induced muscle damage was modified according to a previous study^24^. During exercise, the initial treadmill running speed was set at 5 m/min for 5 min, which was then gradually increased to 10 m/min for 5 min, 15 m/min for 5 min, and 20 m/min for 10 min. Finally, the speed was increased every 3 min at a rate of 2 m/min, to make the mices reach a state of exhaustion. During running, the incline of treadmill belt was set at 8% grade. Exhaustion was determined by two consecutive running failures. For 3 days, imipramine (20 mg/kg) was intraperitoneally injected 1 h prior to the principal exercise (each group N = 8). Blood and gastrocnemius muscle (white: GW, red: GR) were collected at rest (Resting), 0 h (Post-0h), and 24 h (Post-24h) post exercise.

### Western blot analyses

Tissues dissected from the mice were homogenized. The western blot analysis was performed based on the protocol described in our previous study^25^. The optical density of each band was measured using Image J. Anti-SPT-1 (1:300), anti-N-SMase (1:1,000), and β-actin (1:5,000) antibodies were procured from Abcam (Cambridge, MA, USA). Anti-acidic SMase (A-SMase) antibody (1: 500) and anti-caspase-3 antibody were obtained from Santa Cruz Biotechnology, Inc. (Dallas, TX, USA) and Cell signaling Technology (Danvers, MA, USA), respectively.

### Serum CK and IL-6 concentration measurement

The IL-6 and CK levels in serum were determined using a mouse IL-6 ELISA Kit (Ray Biotech, GA, USA) and EnzyChrom^TM^ Creatine Kinase Assay Kit (BioAssay Systems, CA, USA), respectively, according to manufacturer's instructions. The following protocol was followed for measuring the serum CK levels: R1 reagent buffer (100 μl) containing substrate was added to 6 μl of serum sample, and then incubated for 10 min at 37ºC. Following incubation, absorbance was determined at 340 nm wavelength. Serum IL-6 levels were measured as follows: Sample (100 μl) was added into 96-well plate coated with anti-IL-6 antibody and was incubated for 2.5 h at R/T. Next, the sample was incubated with biotin antibody at R/T for 1 h, followed by incubation with streptavidin incubation for 45 min. Absorbance was measured at 450 nm after 30 min of incubation with TMB One-Step Substrate. 

### Statistical analysis

Significant differences between the groups were determined using independent paired *t*-tests and one-way analyses of variance (ANOVA; SPSS for Windows, version 18.0, Chicago, IL, USA). Post-hoc comparisons were made using Fisher’s least significant difference. All values are represented as mean ± standard error (SE), and the statistical significance was set at a value of *p* < 0.05.

## RESULTS

### Acute strenuous treadmill running profoundly enhanced serum CK and IL-6 levels

Serum CK and IL levels were measured to confirm the relevance of the protocol to EIMD model. Serum CK level at 24 h after treadmill running was noted as significantly increased, but not immediately after the exercise (F_2, 24_ = 42.15, *p* < 0.01). Contrastingly, serum IL-6 levels significantly enhanced immediately after exercise, but not 24 h after exercise (F_2, 24_ = 112.68, *p* < 0.01).

### Acute strenuous treadmill running did not affect SPT-1 protein expression in gastrocnemius muscles

To identify the relationship between Cer production by de novo synthesis and EIMD, the expression of SPT-1, which is a key rate-limiting enzyme of de novo synthesis, was measured in gastrocnemius muscles. SPT-1 protein expression did not vary from time to time in gastrocnemius muscles regardless of the time points (for GR: F_2, 9_ = 0.39, *p* > 0.05; for GW: F_2, 9_ = 0.73, *p* > 0.05).

### Acute strenuous treadmill running enhanced A-SMase protein expression of gastrocnemius muscles, but not N-SMase

To identify the relationship between Cer production by sphingomyelin hydrolysis and EIMD, the expression of the two SMases (key rate-limiting enzyme of de novo synthesis) was noted in gastrocnemius muscles. A-SMase protein expression was significantly enhanced between 0–24 h after exercise in both gastrocnemius muscles (for GR: F_2, 9_ =21.28, *p* < 0.01; for GW: F_2, 9_ = 46.05, *p* < 0.01). In contrast, N-SMase protein expression did not change with time in both gastrocnemius muscles (for GR: F2, _9_ = 0.03, *p* > 0.05; for GW: F_2, 9_ = 0.07, *p* > 0.05).

**Figure 1.**
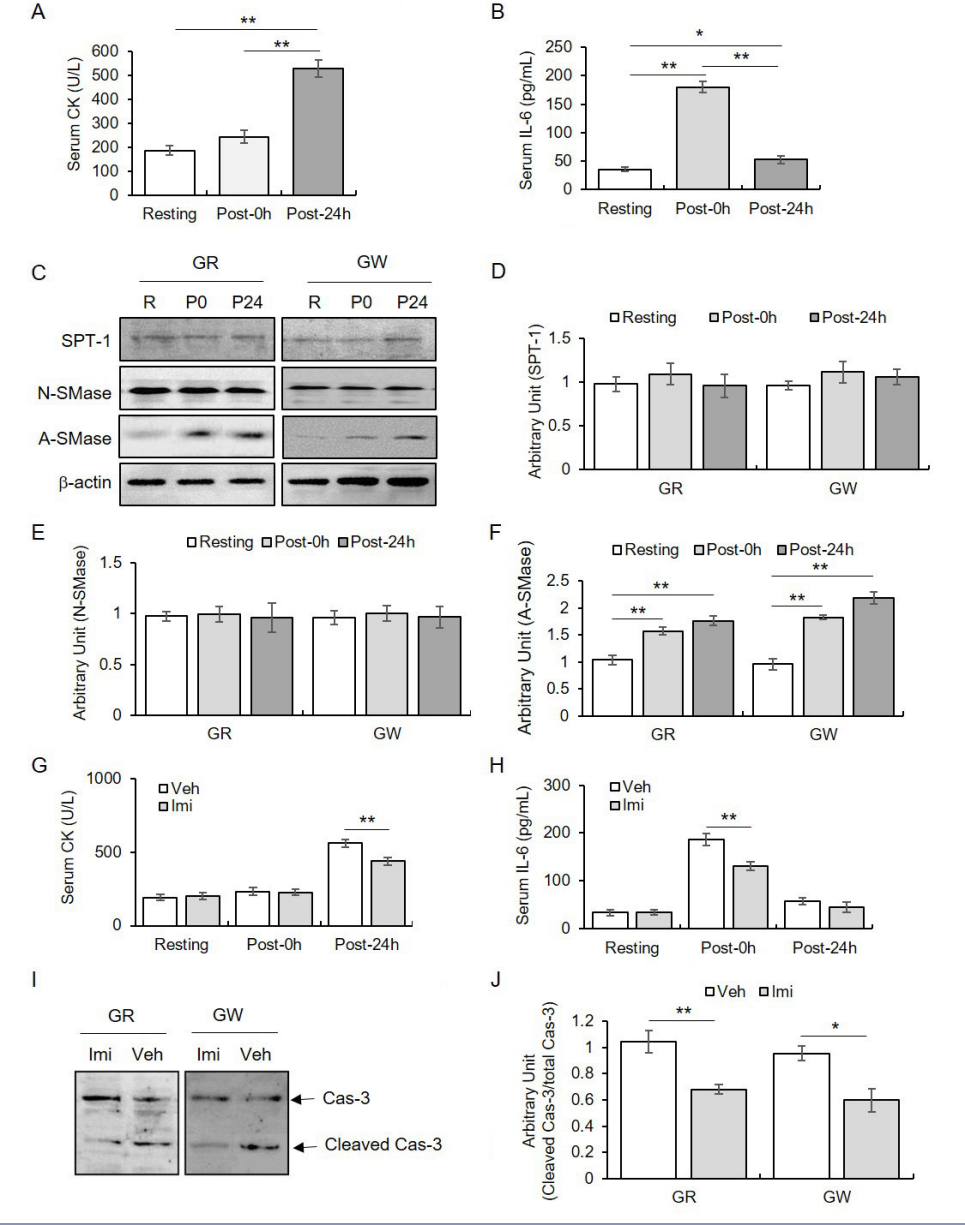
The effect(s) of sphingolipid metabolism on exercise-induced muscle damage. The quantitative analysis of serum (A) CK levels and (B) IL-8 levels in response to strenuous treadmill running, (C) representative images of western blot for SPT-1, N-SMase, and A-SMase. The quantitative analysis of (D) SPT-1, (E) N-SMase, and (F) A-SMase. The quantitative analysis of serum (G) CK levels and (H) IL-6 levels based on imipramine in response to strenuous treadmill running. Representative images of western blot for (I) caspase-3 and the quantitative analysis of (J) caspase-3. Data are presented as mean ± SE. * and ** denote *p* < 0.05 and *p* < 0.01, respectively. CK: creatine kinase; IL-6: interleukin-6; SPT-1: serine palmitoyltransferase-1; N-SMase: neutral sphimgomyelinase; A-SMase: acidic sphigomyelinase; Cas-3: caspase-3; R: resting; P0: immediately after exercise; P24: 24 hours after exercise; GR: red region of gastrocnemius muscles; GW: white region of gastrocnemius muscles

### Imipramine treatment reduced both serum CK and IL-6 levels and cleaved caspase-3 levels in gastrocnemius muscles

The enhanced A-SMase expression in gastrocnemius muscle during EIMD condition led us to explore whether suppressing A-SMase activity reduced EIMD. We found that upon imipramine treatment, serum CK levels significantly reduced 24 h after exercise (for Resting: t_14_ = -0.32, *p* > 0.05; for Post-0h: t_14_ = 0.15, *p* > 0.05; for Post-24h: t_14_ = 4.62, *p* < 0.01), whereas serum IL-6 levels significantly decreased immediately after exercise (for Resting: t_14_ = -0.10, *p* > 0.05; for Post-0h: t_14_ = 4.35, *p* < 0.01; for Post-24h: t_14_ = 1.32, *p* > 0.05).Cleaved caspase-3 levels were significantly reduced in both gastrocnemius muscles 24 h after exercise (for GR: t_6_ = 3.98, *p* < 0.01; for GW: t6 = 3.45, *p* < 0.05).

## DISCUSSION

The current study demonstrated that EIMD might be partly responsible for cellular Cer production by enhancing A-SMase expression against strenuous exercise. Moreover, suppressing A-SMase activity can alleviate skeletal muscle damage in response to intensive exercise.

Strenuous or unaccustomed exercise causes skeletal muscle damage characterized by muscle soreness and decreased muscle function^20-23^. EIMD symptoms depend on the intensity and exercise duration, which can last for several days after exercise^24^. In the present study, serum IL-6 and CK levels represent the systemic indices for muscle damage^26^ and were markedly enhanced immediately (Post-0h) and 24 h (Post-24h) after the cessation of exercise protocol, respectively. This result correlated with the time-course efflux events of EIMD, namely phagocytic and cytotoxic processes, including necrosis and apoptosis. These events occurred and subsequently promoted tissue degradation, beginning with neutrophil-mediated release of pro-inflammatory cytokines^27-33^. Accordingly, the exercise protocol used in the study was sought as appropriate in exploring the role of sphingolipid metabolism in EIMD. As discussed above, eccentric contraction-induced micro-injury of myofibers leads to temporal symptoms and the damaged tissue is eventually regenerated. However, the strategy of reducing EIMD is necessary for rapid recovery between the bouts of physical activity in athletes and leisure club^26,34^. For this reason, the therapy for EIMD, including stretching, massage, electrotherapy, cryotherapy, non-steroidal anti-inflammatory drugs (NSAIDS), as well as nutritional supplementation^26,35-36^ is getting attention in the field of sports. 

Cer, a key molecule of sphingolipid metabolism, contributes to diverse biological processes, including apoptosis, proliferation, differentiation, growth arrest, and inflammation^1-5^. Particularly, cellular Cer promotes apoptosis by activating caspase-3 following stresses such as pro-inflammatory cytokines, and acts as a second messenger of the inflammatory cascade^9-12^. We found that SPT-1 expression in gastrocnemius muscles was not affected at any time points after exercise. SPT-1 catalyzes palmitoyl-CoA condensation with serine to generate 3-ketosphinganine, which is a rate-limiting enzyme of Cer de novo biosynthesis pathway^37^. The result implied that Cer production is not affected through the *de novo* biosynthesis pathway during EIMD. 

In addition to the *de novo* biosynthesis pathway, Cer is also produced by another major pathway, sphingomyelin hydrolysis. SMases that hydrolyze sphingomyelin to Cer and phosphocholine, are divided into the following three categories based on the pH and sub-cellular localization: acidic SMase, alkaline SMase, and neutral SMase^39^. A-SMase and N-SMase are extensively expressed in most tissues, whereas, alkaline SMase is expressed only in the liver and intestine^38^. In the present study, unlike SPT-1, A-SMase levels were enhanced in exercised gastrocnemius (both white and red); however, N-SMase levels were not enhanced. These results suggested that Cer generation may be facilitated through sphingomyelin pathway rather than *de novo* biosynthesis pathway in response to muscle-damaging exercise. This theory was supported by the following evidences: over-expression of A-SMase enhanced TNF-α-induced IL-6 expression^39^. A-SMase-mediated Cer generation induced apoptosis and exacerbated inflammation by serving as a cell surface signaling platform for apoptotic and inflammatory-related receptors such as Fas and TNF-α^40-41^. In other words, EIMD-induced inflammation may induce apoptotic cell death and the secondary inflammation by facilitating A-SMase-mediated Cer production. The induction of A-SMase in gastrocnemius against strenuous exercise led us to determine the strenuous exercise-induced change of serum IL-6 and CK release by inhibiting A-SMase. Notably, exercise-induced increase in serum IL-6 and CK contents were partly reduced by imipramine (a selective inhibitor of A-SMase) treatment. Furthermore, imipramine attenuated strenuous exercise-induced increase in cleaved caspase-3 levels in gastrocnemius. Several previous studies have demonstrated that increased A-SMase expression and activity induced cell death. For example, silica-induced cytotoxicity and inflammation was blocked by inhibiting A-SMase activity in alveolar macrophages^42^. Additionally, oxidized low-density lipoprotein-induced apoptotic cell death was facilitated through the increase in A-SMase activity in smooth muscle cell^43^. The previous findings in combination with the present results suggests that apoptosis of myofibers, which are a component of EIMD, is partly induced by enhancing A-SMase expression and activity. Furthermore, a study exhibited that 5-week of endurance treadmill running enhanced SPT, A- and N-SMase activities of soleus and the gastrocnemius^16^. Although exercise-adapted increase in A-SMase activity was similar to the present data, increased N-SMase and SPT activity were inconsistent. This difference may be attributed to the metabolic energy discrepancy based on the duration and intensity of exercise. Collectively, A-SMase-mediated myofiber cell death after muscle-damaging exercise may be a potential mechanism underlying EIMD. Therefore, inhibiting A-SMase against muscle-damaging exercise may be a good supportive strategy for alleviating EIMD.
